# Tibial Bone Geometry Is Associated With Bone Stress Injury During Military Training in Men and Women

**DOI:** 10.3389/fphys.2022.803219

**Published:** 2022-02-11

**Authors:** Kristen J. Koltun, Nicole M. Sekel, Matthew B. Bird, Mita Lovalekar, Qi Mi, Brian J. Martin, Bradley C. Nindl

**Affiliations:** Neuromuscular Research Laboratory, Department of Sports Medicine and Nutrition, Warrior Human Performance Research Center, University of Pittsburgh, Pittsburgh, PA, United States

**Keywords:** stress fracture, peripheral quantitative computed tomography (pQCT), volumetric bone mineral density (vBMD), musculoskeletal injury risk factor, running, bone imaging, physical training

## Abstract

Bone stress injuries (BSI) are a common musculoskeletal condition among exercising and military populations and present a major burden to military readiness. The purpose of this investigation was to determine whether baseline measures of bone density, geometry, and strength, as assessed via peripheral quantitative computed tomography (pQCT), are predictive of tibial BSI during Marine Officer Candidates School training. Tibial pQCT scans were conducted prior to the start of physical training (*n* = 504; Male *n* = 382; Female *n* = 122) to measure volumetric bone mineral density (vBMD), geometry, robustness, and estimates of bone strength. Bone parameters were assessed at three tibial sites including the distal metaphysis (4% of tibial length measured from the distal endplate), mid-diaphysis (38% of tibial length measured from the distal endplate), and proximal diaphysis (66% of tibial length measured from the distal endplate). Injury surveillance data was collected throughout training. Four percent (*n* = 21) of the sample were diagnosed with a BSI at any anatomical site during training, 10 injuries were of the tibia. Baseline bone parameters were then tested for associations with the development of a tibial BSI during training and it was determined that cortical bone measures at diaphyseal (38 and 66%) sites were significant predictors of a prospective tibial BSI. At the mid-diaphysis (38% site), in a simple model and after adjusting for sex, age, and body size, total area [Odds Ratio (OR): 0.987, 0.983], endosteal circumference (OR: 0.853, 0.857), periosteal circumference (OR: 0.863, 0.824), and estimated bending strength (SSI; OR: 0.998, 0.997) were significant predictors of a BSI during training, respectively, such that lower values were associated with an increased likelihood of injury. Similarly, at the proximal diaphysis (66% site), total area (OR: 0.989, 0.985), endosteal circumference (OR: 0.855, 0.854), periosteal circumference (OR: 0.867, 0.823), robustness (OR: 0.007, 0.003), and SSI (OR: 0.998, 0.998) were also significant predictors of BSI in the simple and adjusted models, respectively, such that lower values were associated with an increased likelihood of injury. Results from this investigation support that narrower bones, with reduced circumference, lower total area, and lower estimated strength are associated with increased risk for tibial BSI during military training.

## Introduction

Musculoskeletal injuries are a significant health concern for the military due to resultant lost duty days, attrition from training, and economic burden (Lovalekar et al., 2021). Stress fractures, in particular, are a relatively common overuse musculoskeletal injury that develop in military personnel with an incidence rate of 5.69 per 1,000 person years (Waterman et al., 2016), and are one of the leading injury causes of lost duty days and repeated training cycles (Cowan et al., 2003). Depending on the site and severity of fracture, rehabilitation can last an average of 27 days up to 12–21 weeks and requires a reduction or cessation of training to support recovery from the injury and time to regain fitness (Wood et al., 2014; Schwartz et al., 2017). Furthermore, stress fractures most commonly affect new recruits compared to more senior service members (Armed Forces Health Surveillance Branch, 2011) and disproportionally affect women (Wentz et al., 2011), thereby impacting military readiness and hindering efforts toward gender integration by interrupting the training pipeline. Of additional long-term concern for warfighter health, and with implications on military operational effectiveness, is the high recurrence rate of stress fractures, such that individuals with a history of stress fracture are more likely to suffer additional fractures in the future (Milgrom et al., 1985). Therefore, due to the prevalence, burden, and long-term effects on military readiness, there is focused emphasis on understanding the pathophysiology and potential risk factors for stress fracture development during military training.

Notably, stress fractures are one component within the broader pathological category of bone stress injuries (BSI), which can exist along a continuum of severity to also include milder stress reactions and complete bone fracture (Pepper et al., 2006; Warden et al., 2014). BSI develop in response to repeated mechanical loading (e.g., running) that produces excessive stress on the bone that outpaces its capacity to heal, ultimately resulting in fatigue failure and the development of microcracks (Pepper et al., 2006). Although bone is a dynamic tissue that is constantly being remodeled through the paired processes of formation and resorption, situations of high intensity, repetitive loading, such as the initiation of military training, can lead to microdamage that will stimulate targeted remodeling to remove damaged tissue, but which may temporarily result in reduced strength due to greater porosity (Robling et al., 2006; Herman et al., 2010). When resorption predominates in the absence of sufficient repair, microdamage accumulates leading to the development of microcracks that result in localized pain and tenderness requiring a reduction in activity to allow for recovery (Robling et al., 2006; Warden et al., 2014). Consistent with the evidence that rapid progressions in mechanical loading can contribute to BSI development, initial military training programs [e.g., Basic Combat Training, Officer Candidates School (OCS)] often result in high rates of BSI (Piantanida et al., 2000; Sulsky et al., 2018). For example, BSI rates are 15–23x higher in recruits compared to more senior service members, and the highest rates were observed among United States Marine Corps (USMC) recruits compared to other branches (26.41 vs. 7.80-15.99) (Armed Forces Health Surveillance Branch, 2011). To date, most investigations assessing BSI risk in Marines have been conducted in non-officer recruits during basic combat training (Beck et al., 1996, 2000; Shaffer et al., 2006; Gaffney-Stomberg et al., 2019). However, BSIs are also a prevalent injury during USMC OCS, with an injury rate of 0.40 and 1.35 injuries per 100 trainees per 1,000 training hours in male and female candidates, respectively, and require the greatest amount of modified training days per injury (Piantanida et al., 2000). As such, OCS presents a novel and valuable opportunity for investigating BSI risk factors in officer candidates during the unique training environment of Marine basic officer training.

The etiology of stress fractures is likely multifactorial and dependent upon combinations of intrinsic and extrinsic components, only some of which may be modifiable. In one study of Army recruits during basic training, stress fracture risk was elevated in association with such factors as female sex, older age, non-Black race, and lower BMI/body mass (Knapik et al., 2012). Additional environmental factors including training program, footwear, and running surface have also been identified (Cowan et al., 2003); however, as most recruits are exposed to similar external stressors during military training, investigations of individual risk factors may be most beneficial for injury screening and incorporation into injury prevention models. One such area of particular interest is bone geometry. Initial military investigations relating bone structure to BSI risk utilized dual-energy x-ray absorptiometry (DXA) and report that reduced diaphyseal dimensions and indices of bone strength were present in Marine recruits with stress fractures compared to those without (Beck et al., 1996, 2000). However, DXA-derived measures of bone geometry and strength are based on two-dimensional estimates that are not of sufficient accuracy to predict individual bone biomechanical characteristics and are therefore limited in their potential clinical utility (Jarvinen et al., 1998; Petit et al., 2005). Alternatively, peripheral quantitative computed tomography (pQCT), which utilizes three-dimensional imaging to measure true volumetric bone mineral density (vBMD) and discriminate between cortical and trabecular compartments, has the capability to directly and accurately measure the geometrical bone structures that underpin bone strength (Petit et al., 2005). Furthermore, pQCT assessments of bone geometry and strength have been associated with stress fractures in runners and military populations (Popp et al., 2009, 2020; Smock et al., 2009; Cosman et al., 2013). For example, in military cadets, despite no difference in areal bone mineral density (aBMD), male cadets with a stress fracture had lower tibial cortical area and bone mineral content than those without a stress fracture (Cosman et al., 2013). Similarly, male and female runners with a BSI had similar tibial vBMD, but lower cortical area and estimated strength compared to healthy controls (Popp et al., 2009, 2020), and male runners with a BSI also had lower total area (Popp et al., 2020). Such findings suggest that measures of tibial bone geometry may provide useful insight for understanding BSI risk during initial military training. Therefore, the purpose of this investigation was to determine whether baseline bone geometry, as assessed by pQCT, is predictive of tibial BSI during USMC OCS military training. We hypothesized that measures of cortical bone geometry at the tibial diaphysis would be associated with tibial BSI development during training such that lower total and cortical area and lower estimated bone strength would be associated with an increased likelihood of BSI.

## Materials and Methods

One pathway of training to be commissioned as an USMC Officer requires completion of OCS, a 10-week initial military training course that consists of intense physical and military training within a controlled and challenging environment. The current investigation is a secondary analysis using data collected as part of a larger study originally designed to identify predictors of injury and resilience in USMC officer candidates. Data were collected during four consecutive iterations of USMC OCS that took place from September 2020 through November 2021. The present investigation utilized data from 504 participants who had complete pQCT, demographic, and injury surveillance data.

During OCS, a candidate’s abilities, performance, and potential as an officer are evaluated according to three tiers consisting of physical fitness, academics, and leadership. Supervised physical training is conducted based on a predetermined schedule and graded events are designed to test general strength and endurance under field and tactical conditions, including completion of endurance courses, ruck marches, and physical fitness tests. For general fitness and to prepare for the graded events, supervised physical training takes place 3–5 days/week and consists of activities including but not limited to running, hiking, body weight exercises, and the completion of obstacle courses. Both male and female candidates complete the same testing and training schedules throughout OCS.

Participants were healthy male and female USMC officer candidate recruits aged ≥ 18 years. All individuals entering the 10-week training course were eligible to participate. Volunteers provided written informed consent prior to participation. This study was approved by the University of Pittsburgh Institutional Review Board and Office of Naval Research (ONR) Human Research Protection Office, endorsed by the OCS Human Research Program, and performed in accordance with the Declaration of Helsinki.

### Study Procedures

This investigation utilized baseline data that was collected following entry to OCS but prior to the initiation of physical training, and injury surveillance was monitored throughout the 10-week training program. Demographics were self-reported on questionnaires and included information regarding age, sex, race, and, for women, menstrual history. Height and weight were measured by trained study staff using a stadiometer and digital scale (Healthometer Professional 500KL, McCook, IL) to calculate BMI.

Prior to the start of physical training, three-dimensional vBMD, bone geometry, and estimated bone strength were assessed via pQCT (XCT2000, Stratec, Germany) at the distal metaphysis (4% of tibial length measured from distal endplate), mid-diaphysis (38% of tibial length), and proximal diaphysis (66% of tibial length) (see Robinson et al., 2019 for illustration of pQCT imaging). Tibial scans were taken of the non-dominant leg, unless orthopedic hardware was present or there was a history of fracture in that limb. The distal metaphyseal (4%) site was selected as a location with predominantly trabecular bone whereas the diaphyseal (38 and 66%) sites were used to investigate cortical bone. Tibial length was measured using an anthropometric tape measure to the nearest millimeter (mm) from the tibial plateau to the medial malleolus. Participants were seated comfortably with their lower leg extended through the gantry and were instructed to sit with their leg still during scanning. Initial scout scans were conducted at a scan speed of 40 mm/s to identify the tibial endplate. Scans of the tibia were conducted at a scan speed of 20 mm/s and a sampling resolution (voxel size) of 0.4 mm. The modes and thresholds utilized for analysis in this study were implemented according to expert recommendations (Bone Diagnostic LLC, Spring Branch, TX). Quality assurance checks were done prior to scanning each day and scan images were assessed following testing. The distal metaphysis (4% site) was assessed for total vBMD, total area, trabecular vBMD, and trabecular area. The mid- (38%) and proximal diaphyseal (66%) regions of the tibia were assessed for total vBMD, total area, cortical vBMD, cortical area, cortical thickness, endosteal circumference, and periosteal circumference. Estimates of bone strength were calculated by computer algorithm for the metaphysis and diaphyseal sites (Bone Diagnostic LLC, Spring Branch, TX). Bone compressive strength was estimated at the distal tibial metaphysis (4% site) as total area*total density^2^ (bone strength index) and bone bending strength was estimated at diaphyseal (38 and 66%) sites as polar moment of inertia of cortical bone area/max distance to center of bone (SSI). Bone robustness was calculated for the metaphysis (4%) and diaphyseal (38 and 66%) sites as total area/tibia length to reflect the relationship between growth in width (area) and total bone length (Jepsen et al., 2013; Popp et al., 2020).

De-identified injury data was acquired from internal OCS records for those who presented to the Physical Therapy department for musculoskeletal injury treatment. All BSIs were diagnosed by trained medical staff based on radiographs or MRI. If subjective history and physical examination suggested a possible BSI, radiographs were performed. If the radiograph confirmed a BSI, no additional radiology was ordered. However, if the initial radiograph was negative or the suspected injury was of a “High Risk” site, MRI were ordered immediately and performed within 48 h. MRIs were read by a radiologist and BSI were diagnosed according to the Fredericson Classification System (Fredericson et al., 1995).

Baseline characteristics are reported as mean and standard deviation (SD) and percentages. To analyze the association between pQCT variables measured at baseline and the risk for future BSI, logistic regression analyses were conducted. Due to the potential contributions of sex, age, and body size on bone parameters and injury risk (Evans et al., 2008; Popp et al., 2009, 2020; Jepsen et al., 2013; Lovalekar et al., 2021) two statistical models were employed. The unadjusted model utilized simple logistic regression to determine whether bone parameters predicted tibial BSI. A second, adjusted, model utilized multiple logistic regression to account for the possible influence of sex, age, and BMI when assessing whether bone parameters predicted tibial BSI during training. To compare differences between those with vs. without a BSI within each sex, independent samples *t*-tests or Mann-Whitney *U*-tests were run. Statistical analyses were conducted with SPSS (version 25.0, IBM Corp, Armonk, NY) and significance was set *a priori* at α ≤ 0.05, two-sided.

## Results

Baseline demographic characteristics are presented in Table 1 and were similar between groups. Participants were 24.9 ± 3.0 years (range: 19–35 years) with a BMI of 25.1 ± 2.3 kg/m^2^ (range: 19.5–33 kg/m^2^). Self-reported race was primarily White (*n* = 362, 72%), followed by Hispanic/Latino (*n* = 69, 14%), Black/African American (*n* = 30, 6%), Asian (*n* = 26, 5%), Other/Not-specified (*n* = 17, 3%).

**TABLE 1:** Baseline demographics of Marine Corps officer candidates.

	Non-BSI			BSI		
	All *n* = 483	Male *n* = 368	Female *n* = 115	All *n* = 21	Male *n* = 14	Female *n* = 7
Age (years)^a^	24.9 ± 3.0	25.0 ± 3.0	24.5 ± 3.2	25.2 ± 2.6	25.5 ± 2.7	24.7 ± 2.7
Height (cm)^a^	173.2 ± 8.2	176.1 ± 6.5	163.9 ± 5.8	171.2 ± 9.2	176.4 ± 5.2	160.9 ± 6.4
Weight (kg)^b^	75.5 ± 10.4	79.0 ± 8.7	64.2 ± 6.8	73.4 ± 11.9	78.7 ± 9.3	62.8 ± 9.6
BMI (kg/m^2^)^b^	25.1 ± 2.2	25.5 ± 2.2	23.9 ± 2.1	24.9 ± 2.3	25.3 ± 2.3	24.2 ± 2.3

*BSI, Bone stress injury. Data are mean ± standard deviation. No baseline group differences observed in the total sample using either Mann-Whitney U-test^a^ or Independent t-test^b^.*

### Bone Stress Injuries

Twenty-one participants (4%) were diagnosed with a total of 27 BSIs during training (Table 2). One female candidate was diagnosed with two BSIs, one male candidate was diagnosed with two BSIs, and one male candidate had five tarsal/metatarsal BSIs. The most common site of injury was the tibia (37%), followed by the tarsals (26%), femur (15%), metatarsals (11%), fibula (7%), and pelvis (4%). Of the 21 participants with a BSI, 13 (62%) were disqualified from training following the injury (Female *n* = 6, Male *n* = 7).

**TABLE 2:** Breakdown of frequency and location of the 27 bone stress injuries that were diagnosed in 21 officer candidates during training.

	All	Male	Female
Tibia	10	6*	4
Tarsal	7	6	1
Metatarsal	3	1	2
Fibula	2	2	0
Femur	4	2	2
Pelvis	1	1	0
Total	27	18	9

*Indicates one injury was categorized as a stress reaction, all others were stress fractures. No injuries were full fractures.

### Bone Density, Geometry, and Estimated Strength

Baseline bone parameters and their association with tibial BSI development during training are presented in Table 3. In both an unadjusted model and after adjusting for sex, age, and body size (BMI), augmented bone parameters at diaphyseal cortical sites were associated with lower likelihoods of developing a BSI. At the mid-diaphysis (38% site), endosteal circumference and periosteal circumference were significantly associated with future BSI such that for every 1-mm decrease, the likelihood of future BSI increased by 14–15 and 14–18%, respectively. Additionally, total area was associated with future tibial BSI such that a 1-mm^2^ decrease was associated with a 1.3–1.7% greater likelihood of BSI and SSI was associated with a 0.2–0.3% greater likelihood of BSI for every mm^3^ decrease. Robustness was only significant in the unadjusted model, in which a 1-mm decrease was associated with a 99.6% greater likelihood of BSI. At the proximal diaphysis (66% site), endosteal circumference, periosteal circumference, and robustness were all associated with future BSI in both statistical models, such that for each 1-mm decrease the likelihood of future injury increased by 15, 13–18, and 99–100%, respectively. Additionally, total area was associated with future tibial BSI such that a 1-mm^2^ decrease was associated with a 1.1–1.5% greater likelihood of BSI, and SSI was associated with a 0.2% greater likelihood of BSI for every mm^3^ decrease. Of note, odds ratios are presented as the change in likelihood of injury for a given one-unit difference in each independent variable and interpretations must incorporate expected real-world variation. For example, a 1 mm decrease in robustness is associated with a twofold change in BSI risk, however, in our sample, the difference in robustness between those with and without a BSI was only 0.1 mm. There were no significant associations among bone outcomes with BSI at the distal metaphysis (4% site).

**TABLE 3:** Tibial volumetric bone density (vBMD), geometry, and estimated strength in Marine Corps officer candidates who developed a tibial bone stress injury (BSI; *n* = 10) during training and those that did not (Non-BSI; *n* = 494).

Predictor	Non-BSI (Mean ± SD)	BSI (Mean ± SD)	OR (95% CI)	*p*-value (Simple logistic regression)	Adjusted OR (95% CI)*	*p*-value (Multiple logistic regression)
Sex (% Female)	118/494 (23.9%)	4/10 (40.0%)	0.471 (0.131, 1.696)	0.249		
BMI	25.1 ± 2.3	24.5 ± 1.7	0.895 (0.676, 1.185)	0.438		
Age	24.9 ± 3.0	25.5 ± 3.3	1.065 (0.877, 1.292)	0.526		

**4%**						

Total vBMD (mg/cm^3^)	345.6 ± 42.2	342.3 ± 38.8	0.998 (0.983, 1.013)	0.806	1.002 (0.986, 1.019)	0.781
Total area (mm^2^)	1170.6 ± 177.5	1059.3 ± 140.5	0.996 (0.992, 1.000)	0.052	0.996 (0.991, 1.001)	0.108
Trabecular vBMD (mg/cm^3^)	285.8 ± 36.5	284.9 ± 35.5	0.999 (0.982, 1.017)	0.936	1.005 (0.987, 1.025)	0.571
Trabecular area (mm^2^)	934.1 ± 156.2	844.7 ± 110.7	0.996 (0.991, 1.000)	0.075	0.996 (0.991, 1.001)	0.152
Bone strength index (mg^2^/mm^4^)	141.1 ± 37.2	126.3 ± 31.6	0.988 (0.970, 1.007)	0.215	0.993 (0.969, 1.017)	0.565
Robustness (mm)	3.1 ± 0.4	2.9 ± 0.3	0.268 (0.048, 1.493)	0.133	0.326 (0.045, 2.379)	0.269

**38%**						

Total vBMD (mg/cm^3^)	936.6 ± 53.4	954.8 ± 53.9	1.007 (0.994, 1.019)	0.288	1.007 (0.994, 1.019)	0.298
Total area (mm^2^)	444.7 ± 64.5	393.6 ± 61.0	**0.987 (0.977, 0.998)**	**0.016**	**0.983 (0.969, 0.998)**	**0.025**
Cortical vBMD (mg/cm^3^)	1149.9 ± 28.7	1152.1 ± 23.4	1.003 (0.981, 1.026)	0.805	0.997 (0.974, 1.020)	0.765
Cortical area (mm^2^)	350.3 ± 53.6	317.8 ± 61.1	0.988 (0.976, 1.001)	0.060	0.987 (0.970, 1.004)	0.128
Cortical thickness (mm)	6.4 ± 0.8	6.3 ± 1.0	0.736 (0.318, 1.704)	0.474	0.938 (0.348, 2.529)	0.899
Endosteal circumference (mm)	34.1 ± 4.8	30.8 ± 2.9	**0.853 (0.738, 0.987)**	**0.032**	**0.857 (0.737, 0.995)**	**0.043**
Periosteal circumference (mm)	74.6 ± 5.5	70.1 ± 5.6	**0.863 (0.768, 0.971)**	**0.014**	**0.824 (0.697, 0.973)**	**0.023**
SSI (mm^3^)	2011.4 ± 412.8	1688.8 ± 375.8	**0.998 (0.996, 1.000)**	**0.017**	**0.997 (0.995, 1.000)**	**0.026**
Robustness (mm)	1.2 ± 0.1	1.1 ± 0.1	**0.004 (0.000, 0.571)**	**0.029**	0.002 (0.000, 1.032)	0.051

**66%**						

Total vBMD (mg/cm^3^)	715.9 ± 66.1	749.4 ± 47.6	1.006 (0.999, 1.014)	0.104	1.006 (0.999, 1.014)	0.105
Total area (mm^2^)	631.3 ± 98.1	537.5 ± 73.5	**0.989 (0.982, 0.997)**	**0.004**	**0.985 (0.975, 0.995)**	**0.005**
Cortical vBMD (mg/cm^3^)	1107.2 ± 33.8	1116.0 ± 39.0	1.008 (0.988, 1.029)	0.416	1.004 (0.984, 1.025)	0.696
Cortical area (mm^2^)	374.0 ± 60.6	337.6 ± 50.1	0.989 (0.978, 1.001)	0.062	0.988 (0.973, 1.003)	0.126
Cortical thickness (mm)	5.1 ± 0.7	5.1 ± 0.5	0.900 (0.355, 2.280)	0.824	1.161 (0.418, 3.226)	0.775
Endosteal circumference (mm)	56.5 ± 6.9	50.0 ± 4.2	**0.855 (0.768, 0.952)**	**0.004**	**0.854 (0.761, 0.960)**	**0.008**
Periosteal circumference (mm)	88.8 ± 7.0	82.0 ± 5.8	**0.867 (0.787, 0.955)**	**0.004**	**0.823 (0.719, 0.941)**	**0.004**
SSI (mm^3^)	2995.4 ± 627.1	2458.0 ± 518.9	**0.998 (0.997, 1.000)**	**0.010**	**0.998 (0.996, 1.000)**	**0.013**
Robustness (mm)	1.6 ± 0.2	1.5 ± 0.2	**0.007 (0.000, 0.214)**	**0.004**	**0.003 (0.000, 0.208)**	**0.007**

*Data are presented as descriptive statistics and results of the simple logistic regression (unadjusted) and multiple logistic regression (adjusted for sex, age, and BMI). Bold indicates p < 0.05.*

*SD, standard deviation; BSI, bone stress injury; OR, odds ratio. CI, confidence interval.*

*Adjusted for sex, age, and BMI.

**FIGURE 1:**
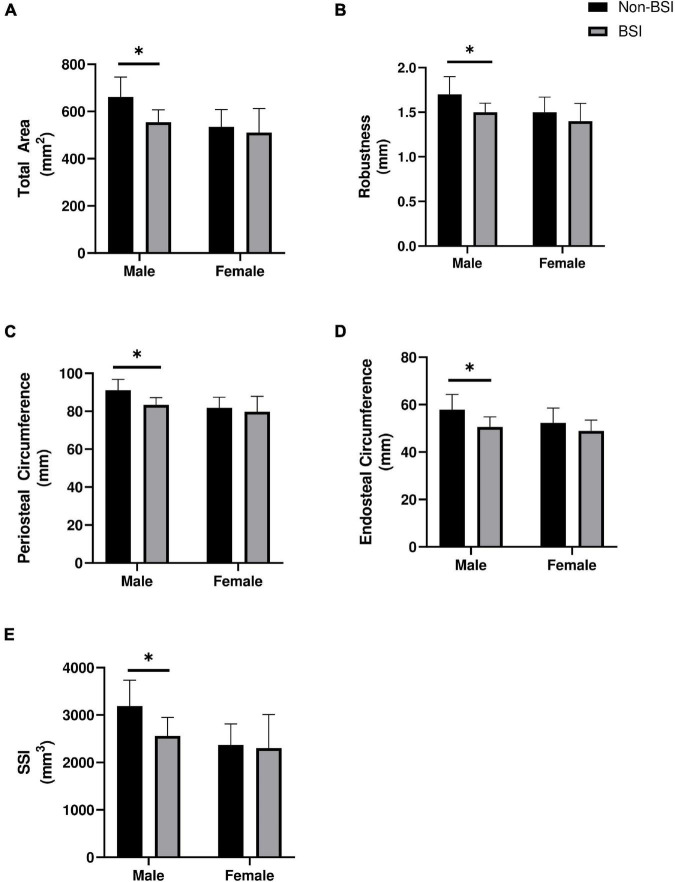
Baseline differences in **(A)** Total area, **(B)** Robustness, **(C)** Periosteal circumference, **(D)** Endosteal circumference, and **(E)** Estimated strength of the proximal diaphysis (66% site) between those who were diagnosed with a bone stress injury (BSI) and those who did not in male (*n* = 6; *n* = 376) and female (*n* = 4; *n* = 118) officer candidates. *Indicates *p* < 0.05. Data are mean ± standard deviation. Mann-Whitney *U*-tests were used to compare estimated strength (SSI) and total area in women and SSI in men, all other comparisons utilized independent-tests.

We further examined whether variables that were significant predictors of BSI in the logistic regression models were different within each sex at baseline (Figure 1). In men, those that were diagnosed with a BSI during training had significantly lower total area (*p* = 0.017, *p* = 0.002), endosteal circumference (*p* = 0.025, *p* = 0.008), periosteal circumference (*p* = 0.015, *p* = 0.002), and estimated strength (*p* = 0.011, *p* = 0.006) at both diaphyseal sites (38 and 66%, respectively). Robustness was significantly lower in men with a BSI compared to those without (*p* = 0.006) at the proximal diaphysis (66% site), but was similar (*p* = 0.065) at the mid-diaphysis (38% site). In women, no significant differences were observed (*p* > 0.05).

## Discussion

This investigation utilized pQCT to examine whether tibial bone density, geometry, and estimated strength were prospective predictors of developing a tibial BSI during OCS military training in male and female USMC officer candidates. Baseline bone parameters including total area, endosteal circumference, periosteal circumference, robustness, and estimated bending strength were all associated with BSI risk such that tibias with reduced circumference, area, and estimated strength were more likely to develop a BSI during training. These findings suggest that baseline pQCT assessments of bone geometry and strength are beneficial for understanding fracture risk in men and women undergoing arduous physical training and, therefore, may have utility for mitigating musculoskeletal injury risk as one component of a risk stratification program.

The primary finding of this investigation was that cortical bone geometry at the diaphyseal tibia (38 and 66% sites) was predictive of prospective BSI during USMC OCS military training. Notably, narrower tibias indicated by lower periosteal circumference, endosteal circumference, robustness, and total area were associated with increased risk of injury both in a simple model and after accounting for sex, age, and body size. Early investigations utilizing DXA and X-ray technology demonstrated that bone size was prospectively associated with stress fracture risk in service members and that those who were injured had bones that were 2–11% narrower compared to the uninjured group (Giladi et al., 1987; Beck et al., 1996, 2000). Additional work utilizing three-dimensional imaging that is able to quantify vBMD and distinguish cortical bone compartments (i.e., pQCT) has further contributed to our understanding of how augmented bone parameters may be protective against BSI risk. In military personnel, injured groups had 6% lower cortical area at the 14 and 33% sites (Cosman et al., 2013; Davey et al., 2015) and 5–7% lower total area and robustness at the 38% site (Jepsen et al., 2013; Davey et al., 2015) compared to those without a BSI. Similar findings have also been reported in runners, an athletic population at elevated risk for BSI, in which men with a history of BSI had 7% lower total area, 9% lower cortical area, and 8% lower robustness at the proximal tibial diaphysis (66% site) (Popp et al., 2020) and women with a history of BSI had 7–8% smaller cortical area, but no differences in total area, at proximal diaphyseal (45, 50, and 66%) sites (Popp et al., 2009). Such differences in total area and robustness are similar to what was observed in the current investigation, which found that those with a BSI had 12.2–16% lower total area and 6.5–8.7% lower robustness compared to the non-BSI group, both of which were associated with greater likelihoods of injury. In contrast to previous reports, cortical area was not predictive of BSI risk in the current investigation; however, most previous investigations did not report endosteal or periosteal circumferences, which may help to explain the non-significant result. In this study, the BSI group had 10.2–12.2% lower endosteal and 6.2–8% lower periosteal circumference compared to the non-BSI group, both of which were predictive of a greater likelihood of injury. Endosteal and periosteal circumferences interact to influence cortical thickness and area; therefore, despite a narrower bone in the BSI group, the greater reduction in endosteal circumference may have preserved cortical area and thickness. With respect to bone density, no measures of vBMD at any tibial site were predictive of BSI during training. Although there are reports of lower aBMD in men and women with BSI compared to healthy controls (Beck et al., 1996, 2000), most investigations have found that aBMD (Cosman et al., 2013), and vBMD (Popp et al., 2009, 2020; Davey et al., 2015) are similar between those with and without BSI.

Consistent with previous literature indicating that slender bones are more susceptible to microcrack accumulation (Hart et al., 2017), the results of this investigation suggest that having a narrow, more slender tibia, which can be indicated by lower total area, robustness, and/or circumference, is a significant predictor of future fracture risk. Firstly, bones with a smaller area may contribute to increased fatigue damage due to overload from higher tissue level stresses since less robust tibias would be expected to experience greater tissue strains and accumulate a greater amount of damage during training (Jepsen et al., 2013). Secondly, there is evidence that tissue-level mechanical properties vary such that narrower bones are more brittle and prone to accumulating damage (Tommasini et al., 2005). Such considerations alone, and in combination, provide insight regarding how bone size and shape may predispose individuals to BSI during arduous physical training.

Notably, Jepsen et al. (2013) demonstrated that bone morphological traits, including cortical area, robustness, and tissue mineral density, may interact to influence tibial stiffness and fracture risk whereby a tibia with higher than expected cortical area or tissue mineral density, but low robustness can be similarly at risk for stress fracture as tibias with average robustness but lower than expected cortical area and tissue mineral density. As such, although individual bone parameters are associated with BSI risk in military and athlete populations, caution should be utilized when interpreting how a single trait can relate to fracture risk. In the current investigation, despite vBMD being similar, differences in geometry did correspond to 17–19.7% lower estimated bone strength at the diaphyseal sites in the BSI group compared to the non-BSI group, which was identified as a significant predictor of future injury. These findings are consistent with those reported in runners, wherein women with a prior lower extremity stress fracture had ∼9% lower estimated bone strength compared to healthy controls (Popp et al., 2009) and, in men, estimated strength was ∼10–16% lower (Popp et al., 2020). Similarly, estimates of bone strength in military populations indicate 11% lower moment of inertia and bending stiffness (Jepsen et al., 2013) and 9% lower SSI (Davey et al., 2015) in those with a BSI compared to non-injured comparison groups. Therefore, bone strength indices that incorporate density and/or geometrical underpinnings may provide greater insight for understanding the complex nature of stress fracture risk rather than assessments of a single morphological trait, such as area or circumference, alone. Findings to date are relatively consistent regarding a role of bone geometry, size, and strength relating to BSI risk in military and athletic populations; however, subtle variations in results are evident and direct comparisons among studies can be difficult due to differences in the technology utilized, the specific sites measured, and the data reported. As bone content, composition, and mechanical integrity can vary along the length of a long bone, measurements and results at different sites along the length of the tibia are not always interchangeable.

Due to the prospective design of this study, data was dependent on the number and location of BSIs diagnosed during training. Incidence rates of BSIs during military training can vary widely depending on country of origin, branch of service, length of training program, and the years surveilled, but the frequency observed in this investigation is within the expected values (Armed Forces Health Surveillance Branch, 2011; Wentz et al., 2011). We observed that 4% (*n* = 21) of our sample were diagnosed with a BSI during USMC OCS training accounting for a total of 27 total injuries, 10 of which (2%) were tibial BSIs.

In this sample, there were no baseline differences in demographic or anthropometric variables between those who developed a BSI during training and those that did not. In military populations, BMI is a well-documented risk factor for musculoskeletal injury (Hruby et al., 2016; Robinson et al., 2016; Jones et al., 2017; Lovalekar et al., 2021), although several previous investigations report similar body weight and BMI between those with stress fracture and healthy control groups (Armstrong et al., 2004; Cosman et al., 2013; Jepsen et al., 2013; Davey et al., 2015). Similarly, female runners did not differ in weight or BMI (Popp et al., 2009); however, male runners with a history of stress fracture were lighter and had lower fat free mass compared to a healthy comparison group (Popp et al., 2020). Interestingly, previous work in healthy young men has demonstrated that lean mass was a positive determinant of bone size, whereas fat mass was associated with smaller bone size (Taes et al., 2009). Therefore, assessments of body composition, rather than total mass, may have greater implications for understanding bone health and injury risk. Additionally, weight loss or changes in body mass may also be relevant for understanding injury risk, as those who developed a stress fracture during Naval Academy training lost more than 4x as much weight as non-injured controls (Armstrong et al., 2004). Unfortunately, multi-compartment assessments of body composition, such as DXA, were not available for this investigation, nor was data collected at the time of injury/attrition to allow for determination of potential weight loss.

Female sex has often been indicated as a risk factor for BSI in military and athlete populations (Wentz et al., 2011; Knapik et al., 2012). In the current investigation, however, sex was not identified as a significant predictor of BSI during training, which may be due to the low sample size of women within the BSI group (*n* = 4) and which may also explain the lack of significant differences in baseline bone parameters between injury groups. In general, women tend to have bone parameters that may predispose them to tibial BSI compared to men, including lower cortical area, total area, tibial diameter, and strength estimates (Evans et al., 2008), and in our sample, both injured and non-injured groups had total area, periosteal circumference, and estimated strength values lower than those of men who developed a BSI. Notably, there are also additional considerations among women that can influence bone health and BSI risk. For example, factors relating to the underlying hormonal milieu, such as menstrual status and hormonal contraceptive use, have been investigated for their association with BSI (Barrow and Saha, 1988; Cobb et al., 2007; Kelsey et al., 2007; Barrack et al., 2014). In this study, none of the women with a diagnosed BSI had delayed menarche, but two did report a history of menstrual irregularity, which is associated with increased risk of BSI (Barrack et al., 2014; De Souza et al., 2014). Three participants were using hormonal contraceptives that mask regular cyclical hormonal fluctuations and preclude assessments of menstrual status, but the fourth self-reported eumenorrheic cycles of ∼26–35 days. No biochemical assessments of hormone status were conducted to confirm status or identify subclinical perturbations and much additional work is required to better understand the unique contributions of endogenous and exogenous hormone exposure to musculoskeletal injury risk in women. Despite sex not being a significant predictor of BSI in our sample, the proportion of women (5.7%) who were diagnosed with BSI during training was higher than that of men (3.7%). Indeed, injuries, particularly BSI, present a major, potentially preventable, cause of modified duty days, and attrition from training for women. As approximately 80% of female candidates are injured during USMC OCS, of which BSI are the most prevalent and occur at a rate that is 3.3x higher than that of male candidates (Piantanida et al., 2000), efforts to assess injury risk factors in female officer candidates are warranted to better maintain the training pipeline and improve graduation success rates.

Strengths of this investigation include the prospective design that included men and women in the sample, and which allowed for all BSI to be diagnosed in the same manner by the same trained medical staff. Additionally, due to the military training environment, all participants were undergoing the same physical training and living under the same conditions thereby controlling for potential external confounders such as exposure, volume of exercise and/or gear requirements. Limitations of this study include that, due to the prospective design, the amount and location of injuries observed were dependent upon the incidence rate. Furthermore, because diagnosis would likely remove them from training, it is possible that some individuals may have had a BSI that they did not seek treatment for and thus would be included in the non-BSI group. Additional analyses of body composition, muscle strength, and bone metabolism may also have been beneficial to provide insight to the etiology and pathophysiology of BSI.

Results from this investigation support the notion that less robust tibia, with lower circumference, area, and estimated strength, are associated with increased risk for tibial BSI and highlight the importance of assessing factors beyond BMD to understand fracture risk. We found that baseline measures of total area, periosteal circumference, robustness, and estimated strength at the diaphyseal tibia were all significant predictors of prospective tibial BSI during military training such that narrower, less robust bones with lower estimated strength were associated with an increased likelihood of injury. Slight differences in bone structure and strength among otherwise healthy young individuals provide important insight regarding injury susceptibility during arduous physical training when fatigue loads are applied in a timeframe that is inadequate for bone recovery and adaptation. As musculoskeletal injuries present a potentially preventable threat to military readiness, injury prevention and prediction initiatives are expanding in an effort to mitigate injury risk and improve resiliency. The leveraging of scientific technologies to assess individual trait characteristics that are related to musculoskeletal injury risk presents a promising avenue for incorporation into screening assessments for maintaining warfighter health. The identification of individuals who may be predisposed to injury presents an opportunity for intervention prior to injury development, such as efforts to optimize bone health prior to arrival at the training environment, or the incorporation of modified physical training schedules, dietary/pharmacological supplementation, and/or equipment adjustments during the training program. Furthermore, multivariate prediction models are likely of greatest value to injury mitigation efforts and the current investigation allows for downselecting evidence-based criterion (e.g., cortical strength estimates) for potential incorporation into more advanced algorithms to assess musculoskeletal injury risk. As the medical field shifts toward personalized, data-driven medicine, the use of biomarkers to inform optimal treatment and training strategies are warranted and three-dimensional bone imaging presents a relevant, field-expedient assessment of BSI risk factors that may have utility as part of risk stratification protocols.

## Data Availability Statement

The datasets presented in this article are not readily available because de-identified participant data is available by application and submission of the proposed use of the data to the study Principal Investigator (BN), which will be considered. Requests to access the datasets should be directed to BN, bnindl@pitt.edu.

## Ethics Statement

The studies involving human participants were reviewed and approved by the University of Pittsburgh and Office of Naval Research. The patients/participants provided their written informed consent to participate in this study.

## Author Contributions

KK was involved with data collection, analyses, and writing. NS was involved with data collection, writing, and manuscript review. MB was involved with data collection, study organization, and manuscript review. ML was involved with data cleaning, analyses, and manuscript review. BM was involved with funding acquisition, study design, data collection, and manuscript review. QM was involved with data cleaning and manuscript review. BN was involved with funding acquisition, study design, and manuscript review. All authors have approved the final version of the manuscript.

## Conflict of Interest

The authors declare that the research was conducted in the absence of any commercial or financial relationships that could be construed as a potential conflict of interest.

## Publisher’s Note

All claims expressed in this article are solely those of the authors and do not necessarily represent those of their affiliated organizations, or those of the publisher, the editors and the reviewers. Any product that may be evaluated in this article, or claim that may be made by its manufacturer, is not guaranteed or endorsed by the publisher.
